# Primary lymphedema of breast, a case report

**DOI:** 10.1016/j.ijscr.2021.106638

**Published:** 2021-11-30

**Authors:** Lana R.A. Pshtiwan, Zuhair D. Hammood, Abdulwahid M. Salih, Zhwan A. Rafaat, Sasan M. Ahmed, Hiwa O. Abdullah, Fahmi H. Kakamad

**Affiliations:** a Smart Health Tower, Madam Mitterrand Street, Sulaimani, Kurdistan, Iraq; b College of Medicine, University of Sulaimani, Madam Mitterrand Street, Sulaimani, Kurdistan, Iraq; c Kscien Organization, Hamdi Str, Azadi Mall, Sulaimani, Kurdistan, Iraq

**Keywords:** Lymphedema, Lymphatic disorder, Breast cancer

## Abstract

**Introduction:**

Lymphedema affects the extremities of breast cancer patients post-surgical or radiation therapy. This study aims to report a case of primary lymphedema of breast.

**Case presentation:**

A 41-year-old female presented with right breast swelling. It associated with hotness and intermittent mastalgia for the past 8 years. Ultrasound (US) examination showed diffuse trabeculate, skin thickening and edematous with normal glandular tissue. Magnetic resonance imaging (MRI) showed diffuse homogeneous parenchymal enhancements with diffuse tranecular and skin edema (high signal on Short-TI Inversion Recovery (STIR)). The result of the biopsy showed normal breast tissue and lymph node.

**Discussion:**

The major risk factors of lymphedema are breast cancer surgery, radiation therapy, axillary lymph node dissection, length and location of breast incision, taking biopsy, trauma, wound infection. Forearm is the most common site of swelling. Primary lymphedema of the breast is a very rare condition without a known risk factor.

**Conclusion:**

Although it is rare, primary lymphedema can affect the breast. US and MRI are necessary to exclude other pathologies. It is managed conservatively.

## Highlights

• Lymphedema mainly develops in breast cancer patients post-operatively.
• Usually affects the extremities and rarely occur in the breast
• It is characterized by the accumulation of extracellular fluid.
• In this paper, a case of breast lymphedema has been discussed.

## 1 Introduction

Lymphedema is a circulatory lymphatic disorder characterized by the accumulation of proteinaceous fluid in the extracellular matrix of adjacent tissues due to insufficiency of the lymphatic system to transport out the lymph fluid in the affected area [1]. This condition may occur as an acute and chronic patterns, which associates with an observable and gradually swelling causing discomfort [2]. As the earlier symptoms of lymphedema, little changes can be observed. Furthermore, clinical signs change according to the duration and severity of the condition [3]. The most common site of initial swelling is the forearm without hand involvement at some circumstances, and may also occur in the axilla, breast, scapular region. Occurrence of breast and chest wall lymphedema without arm swelling is a rare condition [3]. Women with a history of breast cancer surgery or radiation treatment are more suspectible to develop lymphedema. In addition, the most regarded risk factors include axillary lymph node dissection, length of breast incision and location, biopsy of breast, trauma and wound infection [3], [4]. Despite its physiological impacts, lymphedema can cause many physical and psychological side effects such as quality of life, loss of self-confidence, anxiety, depression, limitation of movement and pain [5], [6]. In most of the cases, the condition occurs almost during the first year of post-management period and sometimes delays to decades later [7]. More than 15% of breast cancer survivors are susceptible to face the consequences of lymphedema and the problem is that, no clinical features exist to pre-determine the proportion of patients that would be affected by lymphedema, which may later lead to infection, cellulitis, lymphangitis and even life-threatening septicemia [2], [7], [8].

This study aims to report a case of primary breast lymphedema. The study has been written in line with SCARE guidelines 2020 [9].

### 1.1 Patient information

A 41-year-old female presented with right breast swelling with induration and increase in size for eight years. It associated with hotness and intermittent pain. There was no discharge. Past medical, surgical, drug, and family history was negative.

### 1.2 Clinical findings

**Fig. 1:**
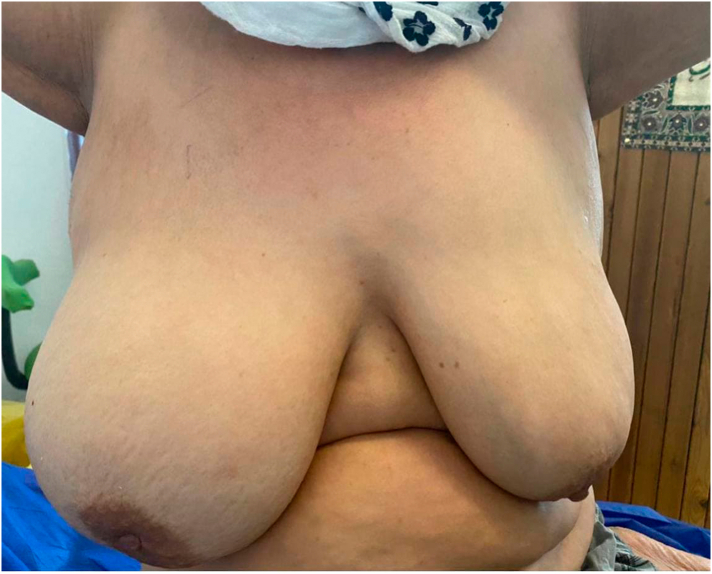
Clinical appearance of both breasts showing asymmetry (large right breast).

Physical examination revealed breast asymmetry, enlarged right breast, Peau d'orange appearance on the skin, normal nipple areola complex, hot on palpation with thickened skin, there was no signs of inflammation (Fig. 1). Both axillae and left breast were normal.

### 1.3 Diagnostic assessment

**Fig. 2:**
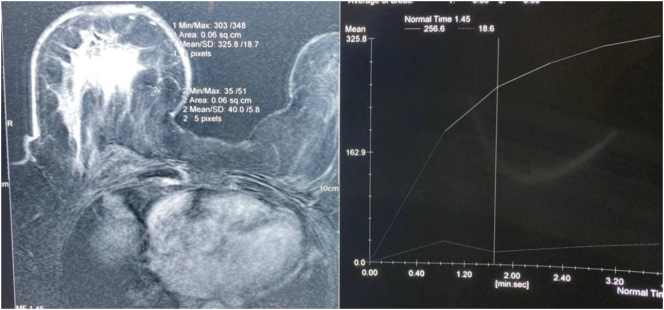
Magnetic resonance imaging (MRI) with plotting showing diffuse homogeneous parenchymal enhancements with diffuse tranecular and skin edema (high signal on Short-TI Inversion Recovery (STIR)), after plotting Total Ion Chromatogram (TIC) to the enhanced parenchyma, showing initial and persistent raising delay phase (type I curve).

US examination revealed diffuse trabeculate, skin thickening and edematous with normal glandular tissues. These were the findings for the last 8 years on repeated US examination. Mammography did not show micro or macro-calcification. MRI showed asymmetrical breast size, the right breast was larger than the left one and displayed a diffuse homogeneous parenchymal enhancements with diffuse tranecular and skin edema (high signal on Short-TI Inversion Recovery (STIR)), no suspicious mass, after plotting Total Ion Chromatogram (TIC) to the enhanced parenchyma, it showed initial phase and persistent raising delay phase (type I curve), no lymphadenopathy could be detected (Fig. 2). True-cut biopsy revealed non-proliferative fibrbocystic breast tissue, negative for malignancy.

### 1.4 Therapeutic intervention

The patient was managed conservatively in form of good hygiene, massage, tight bra, avoiding obesity and acetaminophen tablet 500 mg on need.

### 1.5 Follow up

The patient was followed up for seven years, the swelling fluctuated increasing and decreasing in size.

## 2 Discussion

Lymphedema is a lymphatic disorder causing persistent swelling through abnormal accumulation of fluid in a specific body area [10]. It causes physical and psychological side effects like discomfort, emotional compromising, loss of self-confidence, anxiety, depression, deformity, limitation of movement and pain [5], [6]. There are two types of lymphedema, primary lymphedema when specific cause cannot be found, while in secondary lymphedema, there is an underlining cause. The latter can be caused by breast operation, radiation, axillary lymph node dissection, trauma, infection [5], [6]. Forearm is the most common site of secondary lymphedema [3]. The current case was a 41-year-old women with a history of breast swelling without involvement of any other area. She had intermittent mastalgia for the past 8 years with no mass and discharge. No underlining causes were identified, so it assigned as a case of primary lymphedema of the breast.

The exact prevalence of secondary lymphedema in breast cancer patients after receiving of therapy (chemoradiotherapy, or operation) cannot be easily determined [2]. A study reported that in a short follow up (one year) the incidence following breast cancer treatment decreased and a long-term follow up (reaching 11 years) showed increase the the incidence. [11]. According to data of seven international surveys since 1990, there was a great variation in incidence from 6% to 30%. It is unknown yet why this disorder appears only in a fraction of the patients after breast cancer treatment, while others are immune from this complication [12]. In the present study, the patient had only a little pain that did not affect the quality of life and daily activity.

Lymphedema could be acute or chronic. Acute lymphedema is usually mild and appears post operatively within a few days, it forms a pitting edema subsiding after several weeks. If it would be left without proper treatment it would progress to chronic lymphedema. It is characterized by turning the skin to brawn and fibrotic in moderate situations. While in severe circumstance, the accumulation of proteinaceous fluid causes pain, numbness, mobility restriction, loss of skin elasticity. [12]. The condition in the current case was chronic with mild symptoms.

Lymph stasis is an adverse outcome of long-period inflammation, which may end with vessel sclerosis that is resistant to treatment. So, regardless to the type of lymphedema (arm, breast), it is crucial to precisely monitor the incidence after surgery [13]. Various modalities are available to diagnose lymphedema such as MRI, volume measurement by water displacement and geometrical calculation, Lymphoscintegraphy, multiple frequency bioelectrical impedance analysis (MFBIA). Furthermore, MRI is a quite informative technique for detecting lymph accumulation within the entire limb but it is expensive and time consuming. Using water displacement for measuring limb volume is rarely reliable because of lacking patient consent. Even though, geometrical calculation is a quick and easy technique, but depending on the total volume of limb to determine the increase of extracellular fluid volume decreases its sensitivity [14]. In the present study, US and MRI showed edema and swelling in the right breast with dilated subdermal lymphatic ducts with several normal looking lymph nodes. Fine needle aspiration was negative.

The main treatment modality is conservative measures including massage, compression bandaging, exercise, tight bra and skin care [13], [15]. Lymphovenous anastomosis (LVA), vascularized lymph node transfer (VLNT) and debulking surgery are the common surgical interventions. According to the literature, only a few cases available that have specifically been dealt with breast lymphedema treatment and LVA was the chosen option in all of them. LVA was used for the first time by Yamamoto et al. In 2016 [16] The current case was treated conservatively due to good response.

In conclusion, lymphedema is the excessive accumulation of fluid in the interstitial tissue. Although it is rare, it can occur primarily in the breast.

## Ethical approval

Approval is not necessary for case report (till 3 cases in single report) in our locality.

The family gave consent for the publication of the report.

## Research registration

Not applicable.

## Trial registry number

Not applicable.

## Author contribution

Abdulwahid M. Salh: major contribution tothe idea, literature review, final approval of the manuscript.

Fahmi H. Kakamad, Zuhair D. Hammood: writing the manuscript, final approval of the manuscript.

Lana R.A. Pshtiwan, Zhwan A. Rafaat, Sasan M. Ahmed, Hiwa O. Abdullah: literature review, final approval of the manuscript.

## Guarantor

Fahmi Hussein Kakamad.

## Patient consent

Written informed consent was obtained from the patient for publication of this case report and accompanying images. A copy of the written consent is available for review by the Editor-in-Chief of this journal on request.

## Source of funding

None is found.

## Provenance and peer review

Not commissioned, externally peer-reviewed.

## Declaration of competing interest

None to be declared.
